# Chromo- and fluorophoric water-soluble polymers and silica particles by nucleophilic substitution reaction of poly(vinyl amine)

**DOI:** 10.3762/bjoc.6.79

**Published:** 2010-07-22

**Authors:** Katja Hofmann, Ingolf Kahle, Frank Simon, Stefan Spange

**Affiliations:** 1 Department of Polymer Chemistry, Chemnitz University of Technology, Straße der Nationen 62, Chemnitz 09111, Germany; 2 Leibniz Institute of Polymer Research Dresden, Hohe Straße 6, Dresden 01069, Germany

**Keywords:** carbonitrile, cyclodextrin, fluorescence, hybridmaterials, poly(vinyl amine)

## Abstract

Novel chromophoric and fluorescent carbonitrile-functionalized poly(vinyl amine) (PVAm) and PVAm/silica particles were synthesized by means of nucleophilic aromatic substitution of 8-oxo-8*H*-acenaphtho[1,2-*b*]pyrrol-9-carbonitrile (**1**) with PVAm in water. The water solubility of **1** has been mediated by 2,6-*O*-β-dimethylcyclodextrin or by pre-adsorption onto silica particles. Furthermore, **1** was converted with isopropylamine into the model compound **1-M**. All new compounds were characterized by NMR, FTIR, UV–vis and fluorescence spectroscopy. The solvent-dependent UV–vis absorption and fluorescence emission band positions of the model compound and the carbonitrile-functionalized PVAm were studied and interpreted using the empirical Kamlet–Taft solvent parameters π^*^ (dipolarity/polarizability), *α* (hydrogen-bond donating capacity) and *β* (hydrogen-accepting ability) in terms of the linear solvation energy relationship (LSER). The solvent-independent regression coefficients a, b and s were determined using multiple linear correlation analysis. It is shown, that the chains of the polymer have a significant influence on the solvatochromic behavior of **1-P**. The structure of the carbonitrile **1-Si** bound to polymer-modified silica particles was studied by means of X-ray photoelectron spectroscopy (XPS) and Brunauer–Emmett–Teller (BET) measurements. Fluorescent silica particles were obtained as shown by fluorescence spectroscopy with a diffuse reflectance technique.

## Introduction

Fluorescent polymers are an important tool for the study of molecular dynamics and also find use as sensor probes for biologically active compounds [1]. In recent years, there has been great interest in the synthesis, characterization and applications of fluorescent polymers [2–4]. In general, there are two approaches for the synthesis of fluorescent polymers: First, the polymerization of a monomer that contains a fluorescent chromophore is possible. However, in some cases the new fluorophore-carrying polymer [1,5] is accompanied by a non-adequate effort concerning its synthesis and purification. An alternative pathway is the chemical modification of commercially available polymers containing reactive groups which can react with fluorescent dyes [6]. Poly(vinyl amine)s (PVAm) are copolymers made by the hydrolysis of poly(vinyl formamide) (PVFA). Fully hydrolyzed PVFA contains ca. 5% of formamido groups and 95% of primary amino groups. The water-soluble PVAm copolymer has been widely applied for a number of purposes, e.g., in catalysis [7], chelation [8], treatment of waste water [9], paper making [10], recovery of oil [11] and as superabsorber [12]. Modified PVAm is expected to be useful in the preparation of polymeric water-soluble dyes [13], in mimicking natural enzymes [14] and as a polymeric surfactant [15]. Depending on the pH of the aqueous solution, the primary amino groups of PVAm can be partly protonated (−NH_3_^+^) or they can be present as charge-neutral amines (−NH_2_). In this context, PVAm can be considered as a weak cationic polyelectrolyte.

The pH-variation is an excellent tool to control and adjust the net-charge density along the polymer chain or in a layer made from PVAm [16–17]. Full conversion of the amino groups into their cationic form leads to a polymer with the highest known charge density along the polymer backbone. Such highly protonated polymers appear to be very interesting compounds for the prevention of bacteria adhesion on surfaces [18]. Non-protonated primary amino groups in the PVAm polymer have a high synthetic potential which can be used for versatile subsequent derivatization reactions [17,19–21].

In previous articles we reported the nucleophilic aromatic substitution of activated fluoroaromatic compounds with PVAm [22–24]. Reactions of PVAm with derivatization agents seem at first glance to be simple to carry out, but in fact they require a considerable synthetic effort because the reactants must share a solvent or a homogeneous mixture of solvents. For many reactions it is profitable to remove or to buffer low-molecular mass products formed during the reaction between PVAm and the derivatization agent.

High molecular mass products of PVAm copolymers (*M*_r_ > 1500 g mol^−1^) are soluble in water only, while fluoroaromatic compounds are sparingly soluble or even insoluble in water. The lack of solvents capable of dissolving all the reactants complicates subsequent derivatization reactions or requires a high synthetic effort. Micelle techniques commonly used to carry out reactions between water-soluble and insoluble compounds cannot be cleanly applied to modify polyelectrolytes [25]. Alternatively, water-soluble polymers can be modified by the introduction of alkyl or aryl groups, thus mediating solubility in organic solvents [26].

The use of cyclodextrins (CD) provides a further opportunity to solve these problems. Due to the formation of host–guest complexes with CD both reactants become completely soluble in water. According to Ritter et al., the 2,6-*O*-β-dimethylcyclodextrin (β-DMCD) derivative has been found to be suitable because of its higher solubility in water compared to β-cyclodextrin (800.5 g L^−1^ vs. 18.5 g L^−1^) [27–29].

Chemical reactions between two incompatible reactants can also be achieved under heterogeneous reaction conditions (the reaction is localized at the interphase of the two contacting phases). This approach was developed to carry out functionalization reactions between the water-soluble PVAm and suitable chromophores [30] or fluorophores. In these reactions, silica can be considered as ‘solubilizer’ because PVAm, as well as the reactants, are consecutively adsorbed on the silica particles. The functionalization reactions take place on the silica surface simultaneously. The large specific surface area of the silica particles employed (ca. 400 m^2^ g^−1^) guarantees the presence of a high amount of the adsorbed polymer and modifiers [31].

The electron-deficient heterocycle 8-oxo-8*H*-acenaphtho[1,2-*b*]pyrrol-9-carbonitrile (**1**) is a type of novel fluorescent chromophore with long-wavelength absorption and fluorescence, the excitation and emission wavelengths of which can reach 530 nm and 590 nm, respectively [36]. It is well known, that carbonitrile **1** is able to react with nitrogen-, oxygen-, or sulfur-containing nucleophiles by nucleophilic aromatic substitution reactions (S_N_Ar^H^) [32–36]. The reaction of **1** with 3-thiopropionic acid has used to produce a fluorescent sensor for cys/Hcy with a 75-fold fluorescence enhancement [34]. The S_N_Ar^H^ reaction of **1** with the thiolated RGD peptide cyclo(Arg-Gly_Asp-Phe-Lys(mpa))(c(RGDFK)-SH) leads to a fluorescent sensor for imaging tumor cells [37].

In this paper we report our current studies on the functionalization of PVAm with carbonitrile **1** to introduce a chromophore as well as a fluorophore into the polymer chain. The synthesis was achieved by two different approaches. The first method included the use of β-DMCD to render **1** compatible to PVAm dissolved in water. The S_N_Ar^H^ reaction between the two reactants was carried out in homogeneous phase. The second approach was to synthesize a hybrid material by the consecutive adsorption of PVAm and **1** onto silica particles. The adsorbed substances were reacted directly on the silica particle surface. The reaction products (yield on the surface of silica particles) were analyzed using high-resolution X-ray photoelectron spectroscopy (XPS). The XPS method is very surface-sensitive and gives spectroscopic information for sample depths of less than 8 nm.

## Results and Discussion

### Synthesis

#### Synthesis and characterization of the fluorescent polymer

**Scheme 1:**
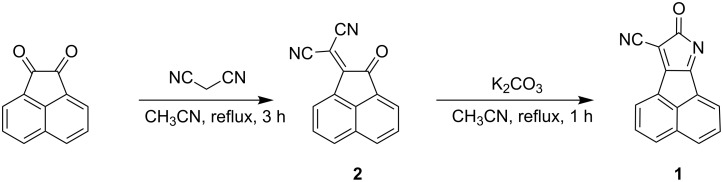
Synthesis of carbonitrile **1**.

Carbonitrile **1** was synthesized by a very efficientl two-step literature procedure [32,34]. As shown in Scheme 1, the starting material acenaphthylene-1,2-dione undergoes a Knoevenagel condensation with malononitrile to give mono-adduct **2**.

Compound **2** was originally synthesized by Junek et al. [38]. However, no detailed NMR data were reported. In a second step, cyclization with anhydrous K_2_CO_3_ converted **2** to **1** in a good yield. In contrast to the procedure described in [34], a ten-fold excess of K_2_CO_3_ did not give rise to the target compound **1**. Only the use of a 10 % mol. equivalent of the base led to **1**.

**Scheme 2:**
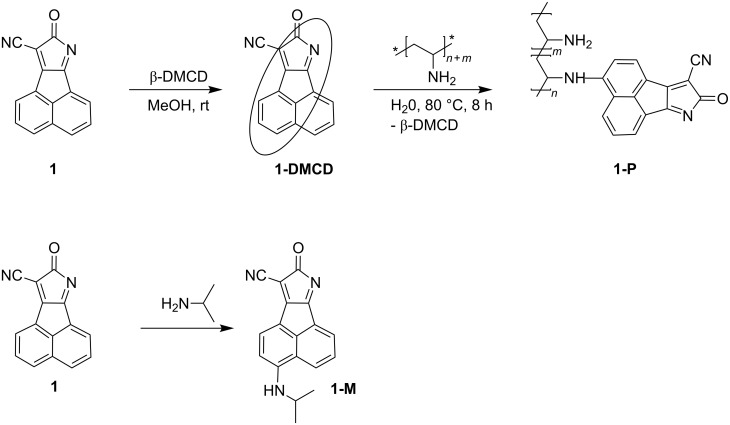
Coupling of the β-DMCD-caged carbonitrile derivative **1-DMCD** with PVAm to yield the fluorophore-functionalized PVAm **1-P** and synthesis of the model compound **1-M**.

The success of the S_N_Ar^H^ reaction of **1** with PVAm (Scheme 2) mediated by β-DMCD in water could be established using ^13^C-{^1^H}-CP-MAS NMR spectroscopy.

**Figure 1:**
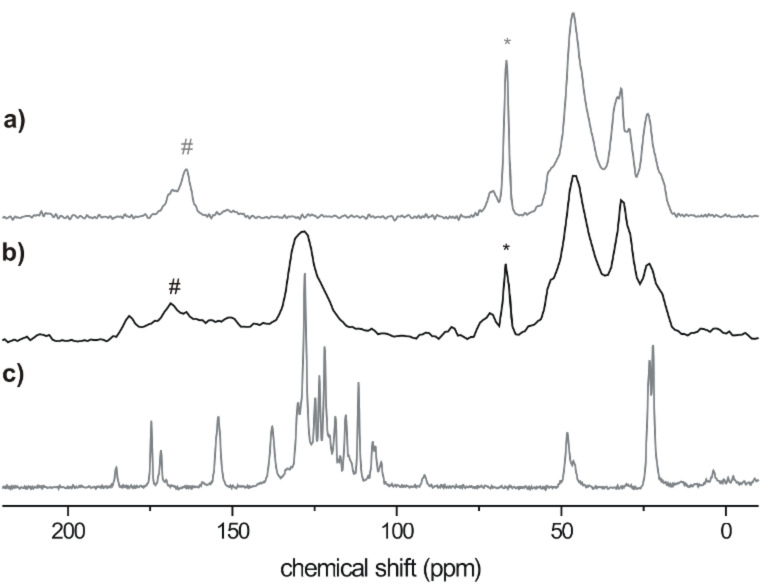
Solid state ^13^C-{^1^H}-CP-MAS NMR spectra of pure PVAm (a), functionalized PVAm **1-P** (b) and the model compound **1-M** (c) [“*” indicates the signals of the methine carbon of poly(vinyl alcohol) (PVA) and “#” corresponds to non-hydrolized formamido groups still present in the PVAm chains].

Figure 1 compares the solid state ^13^C NMR spectra of pure PVAm, fluorophore-functionalized PVAm **1-P** and the model compound **1-M**. It can be seen that the signals observed for **1-M** and pure PVAm are also visible in the ^13^C-{^1^H}-CP-MAS NMR spectrum of **1-P**. This is an excellent evidence for the functionalization of the PVAm fluorophore **1**. The signal at δ = 169 ppm in the solid state ^13^C NMR spectra [(a) and (b), Figure 1] are due to the residual formamido groups while the signal at δ = 67 ppm is caused by the methine carbon of poly(vinyl alcohol) [39–40].

Further confirmation of the success of the S_N_AR^H^ reaction was obtained from the FTIR spectra. Pure PVAm copolymers show characteristic IR absorption of N–H groups in the range of 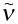 = 3450–3150 cm^−1^ due to the stretching vibration and at 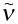 = 1600 cm^−1^ due to the deformation vibration. Asymmetric as well as symmetric stretching modes of C–H groups (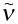 = 2880–2950 cm^−1^) and deformation vibrations of the CH_2_ and CH group at 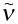 = 1430 cm^−1^ as well as 1381 cm^−1^ were observed. Additionally, the IR band at 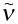 = 1665 cm^−1^ corresponds to residual formamido groups. The IR spectrum of the model compound shows a sharp N–H stretch band at ca. 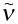 = 3334 cm^−1^, stretching modes of aromatic and aliphatic C–H groups between 2879–3080 cm^−1^, C≡N stretching at 2213 cm^−1^ and aromatic C=C stretching vibrations at 1627 cm^−1^. The IR spectrum of the carbonitrile-functionalized PVAm **1-P** shows the characteristic absorption bands of both the PVAm and carbonitrile moiety which confirms the functionalization. A broad N–H stretching band of the NH_2_ groups of the polymer and the NH group of the fluorophore at 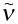 = 3550–3150 cm^−1^ was observed. Bands between 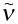 = 2890–2990 cm^−1^ correspond to the stretching modes of the aliphatic CH groups of the polymer backbone. The IR absorption of the aromatic C=C units occurs at ca. 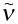 = 1665 cm^−1^ which appear to be overlapped by N–H deformation vibration bands. The presence of the C≡N stretching band at 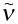 = 2221 cm^−1^ confirms the existence of the organo-substituted fluorophore.

The glass transition temperature *T*_g_ is an important parameter for polymers, which indicates the transition of the amorphous phase of the polymer from its rubbery to its glassy state (and vice versa). Pure PVAm shows a *T*_g_ of 103 °C. The glass transition temperature is a function of chain flexibility. Because of the presence of hydrogen-bond donor and -acceptor centers along the polymer backbone, PVAm is able to form hydrogen bonds between the amino groups (and residual formamido groups). The carbonitrile-functionalized PVAm **1-P** shows a lower *T*_g_ of 87 °C. Obviously, the introduction of the rigid fluorophore leads to a separation of the individual polymer chains, resulting in a decreased glass transition temperature.

#### Synthesis and characterization of the hybrid material

**Scheme 3:**
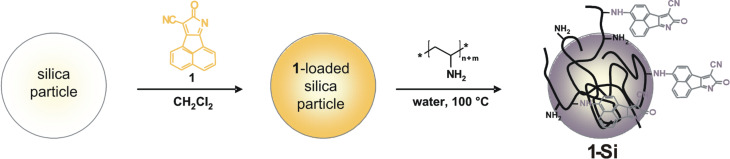
Synthesis of **1-Si** by nucleophilic aromatic substitution of **1** adsorbed onto silica particles.

As can be seen from Scheme 3, carbonitrile **1** was adsorbed from its dichloromethane solution onto carefully dried silica particles (Kieselgel 60, Merck). The **1**-loaded silica particles were transferred into an aqueous solution of PVAm. In order to initiate the S_N_AR^H^ reaction between **1** and PVAm the solution was heated and kept for 16 h at its boiling point. The silica particles were extracted twice, first with water to remove non-adsorbed functionalized PVAm and then with acetone to remove unreacted **1**. **1-Si** was obtained as intensively colored purple solid.

The ^13^C-{^1^H}-CP-MAS NMR spectrum of **1-Si** shows the characteristic signals of the fluorophore as well as those of the aliphatic carbons of the polymer backbone. The FTIR spectrum of **1-Si** confirms the adsorption of the functionalized PVAm onto the silica particle surface. As expected, a strong IR absorption band was present in the range of 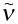 = 1200–1000 cm^−1^ corresponding to Si–O–Si stretching vibrations. Furthermore, a broad N–H stretching vibration at 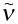 = 3150–3500 cm^−1^ was observed. Additionally, the IR spectrum shows stretching vibrations at 2207 cm^−1^, 1651 cm^−1^ and 1372 cm^−1^ (ν_C≡N_, ν_C=C_ and δ_CH_) which are evidence for the adsorption of carbonitrile-functionalized PVAm.

**Figure 2:**
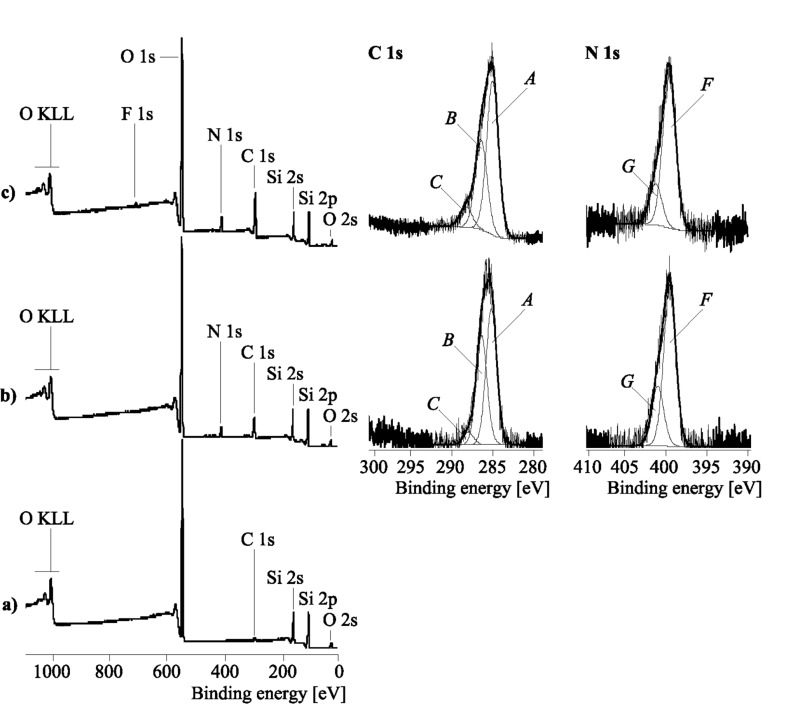
Wide-scan X-ray photoelectron spectra (left), C 1s and N 1s high-resolution spectra (right) of bare silica particles (a), PVAm adsorbed onto silica particles (b) and PVAm reacted with **1** on the surface of silica particles (c).

XPS spectra were taken from the unmodified silica, PVAm adsorbed onto silica and the fluorophore PVAm-modified silica particles **1-Si** (Figure 2).

Figure 2a shows a typical XPS wide-scan spectrum of the bare silica support which was unloaded. The spectrum contains the expected peaks from silicon (Si 2s and Si 2p) and oxygen (O 1s, O 2s and the O KLL Auger series). The small C 1s peak shows the presence of typical hydrocarbon surface contaminations. PVAm, adsorbed onto the silica particle surface, strongly increases the amount of carbon on the silica surface. Nitrogen atoms of the amino (and residual formamido) groups lead to the N 1s peak (Figure 2b). The corresponding C 1s high-resolution spectrum of the PVAm-loaded silica shows saturated hydrocarbons from the methylene units of the polymer backbone and surface contaminations (component peak *A*). Component peak *B* shows C–N bonds of primary amino groups of the polymer and the amine-side carbon atoms (O=CH–NH–C) of residual amido groups. Formamido groups (O=CH–NH–C) contribute to the component peak *C*. The area of component peak *C* is ca. 4.9% of the C 1s area. As noted above, the amino groups of the PVAm polymer can be protonated by hydronium ions. In Figure 2b the N 1s high-resolution spectrum of the PVAm/silica hybrid material shows such protonated amino groups (component peak *G*), clearly separated from the non-protonated amino groups (component peak *F*). Nitrogen involved in amide groups contributes to component peak *F*. The ratio of the two component peak areas [*G*]:[*F*] can be considered as the protonation–deprotonation equilibrium of the amine-functionalized hybrid surface.

The reaction of adsorbed PVAm with adsorbed **1** changes the corresponding XPS spectra slightly (Figure 2c). In the wide-scan spectrum the amounts of carbon and nitrogen increase: The elemental ratio [N]:[O] of the PVAm loaded silica sample was found to be [N]:[O] = 0.094, while the ratio after reaction of PVAm with **1** was [N]:[O] = 0.152 (nitrogen can be considered as label for the organic adsorption layer and oxygen the label for the silicon support). The coupling of PVAm with the fluorophore group **1** introduces an additional amount of carbonyl groups (carbon atoms of the cyclic amide in structure **1-P**) into the organic adsorption layer. Hence, in the corresponding C 1s spectrum (Figure 2c) the area of component peak *C* is increased to 8.11%. Carbon atoms of the C≡N group, C–N bonds in structure **1** and C–NH–C links between **1** and the PVAm polymer contribute to component *B*. The attachment of the fluorophore to the PVAm also changes the ability of the nitrogen atoms to be protonated. Nitrogen atoms in structure **1** cannot be involved in the protonation–deprotonation equilibrium of the amino groups of the PVAm polymer. The N 1s spectrum in Figure 2c shows a significantly decreased area of component peak *G* which is due to the C–N^+^ species (its binding energy is BE ≈ 401.4 eV while the binding energy of the non-protonated species is BE ≈ 399.5 eV).

**Figure 3:**
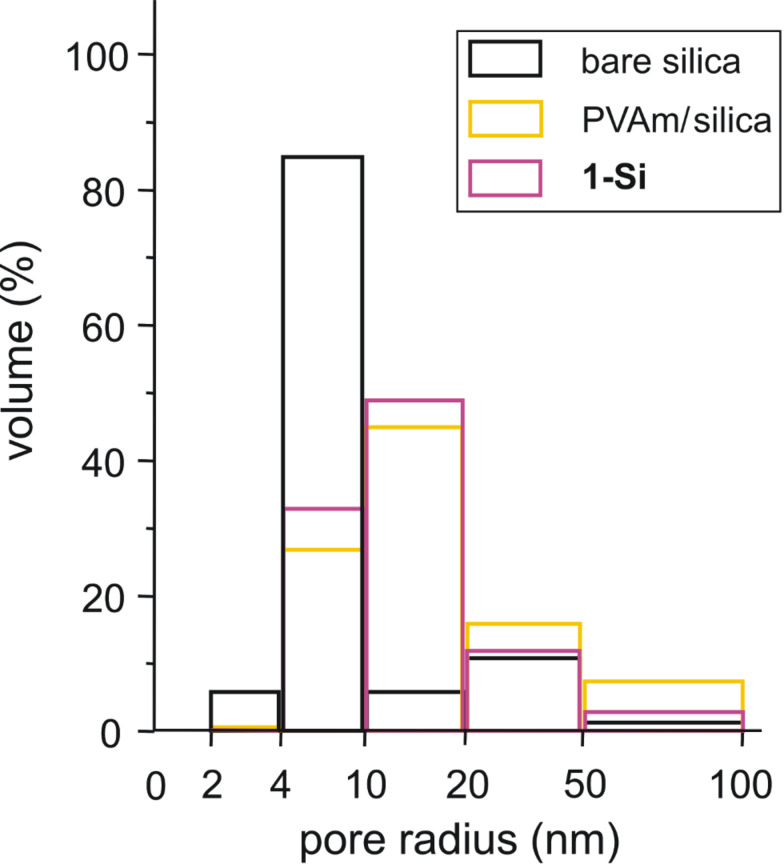
Pore-size distribution of bare silica, PVAm/silica and **1-Si**.

Table 1 summarizes the results of the BET analysis. In addition, Figure 3 shows the pore-size distribution histograms of bare silica, a typical PVAm/silica hybrid material and **1-Si**.

**Table 1:** Specific surface areas (according to BET, A_BET_) and fractions of the average pore-size radii range of the bare silica support, a PVAM/silica hybrid material and **1-Si**, compared with the relative carbon-content (C_found_/C_calc_·100%) determined from data of the elemental analysis.

sample		Fraction of the average pore radii (%)		C_found_/C_calc_
	A_BET_ (m^2^ g^−1^)	2–4 nm	4–10 nm	100%

bare silica	411	85	6	–
PVAm/silica	207	27	45	75.50
**1-Si**	197	0.3	33	89.05

The bare silica support used for the functionalization reaction showed a BET surface of 411 m^2^ g^−1^. The main average pore radius ranges between 2 and 4 nm. The adsorption of PVAm as well as **1-Si** significantly reduces the specific surface area. Small molecules such as the fluorophore **1** preferably fill the narrow pores. Hence, the fraction of the narrow pore radii 2–4 nm is considerably decreased. PVAm also reduces this. But the macromolecules are also able to cover the porous silica surface and prevent the access of the BET probe molecules (nitrogen) to the inner silica surface. The corresponding BET measurements show a smaller value of specific surface area (Table 1). After the adsorption of **1** or PVAm the fraction of the pore sizes between 4 and 10 nm is apparently higher. However, the values given in Figure 3 are expressed in percent terms. The increased values profit from the decreased value of the accessible narrow-size pores.

### Solvatochromic and fluorescence properties

Interactions of solvatochromic dyes with pure solvents or solvent mixtures are a combination of many effects [41–44]. We wanted to investigate the influence of the PVAm on the chromophoric and fluorophoric π-electron system of **1** resulting from intermolecular interactions with the surroundings of the molecules, and which of these are dipole–dipole and/or hydrogen-bond interactions. To separate the effects of non-specific van der Waals interactions including electrostatic effects (dipolarity/polarizability) from specific interactions (hydrogen bonding), we used the simplified Kamlet–Taft equation (Equation 1) [45–46].

[1]
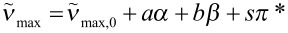


According to Equation 1, the influence of the hydrogen-bond donor capacity (HBD) [47], the hydrogen-bond acceptor capacity (HBA) [48] and the dipolarity/polarizability [45,49] of a solvent can be expressed by *α*, *β* and π^*^, respectively. 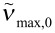 corresponds to a standard process, referenced to a nonpolar medium. a, b and s represent solvent-independent regression coefficients which reflect the relative influence of each of the three parameters.

#### UV–vis spectroscopy

The solvatochromism of **1-M** and **1-P** was investigated in a set of only ten solvents due to the low solubility of **1-P** in organic solvents. Table 2 shows the UV–vis absorption maxima of **1-M** and **1-P** measured in solvents of different polarity and hydrogen-bonding ability, and the Kamlet–Taft parameters used for the multiple linear correlation analysis.

**Table 2:** UV–vis absorption maxima, 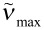, of **1-M** and **1-P** investigated in ten solvents of different polarity and hydrogen-bond ability and the empirical Kamlet–Taft parameter *α*, *β* and π^*^ [41,43].

Solvent	Kamlet–Taft parameters			**1-M**		**1-P**	
	*α*	*β*	π^*^	λ_max_ (nm)	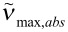 (10^3^ cm^−1^)	λ_max_ (nm)	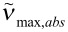 (10^3^ cm^−1^)

DMF^a^	0	0.69	0.88	578	17.30	534	18.73
DMAA^b^	0	0.76	0.88	579	17.27	535	18.69
DMSO^c^	0	0.76	1.00	580	17.24	532	18.79
DCM^d^	0.13	0.10	0.82	571	17.51	— ^g^	— ^g^
acetonitrile	0.19	0.41	0.75	573	17.45	531	18.83
1-propanol	0.84	0.90	0.52	575	17.39	536	18.66
ethanol	0.86	0.75	0.54	574	17.42	538	18.59
methanol	0.98	0.66	0.60	575	17.39	535	18.69
TFE^e^	1.51	0	0.73	569	17.57	521	19.19
HFIP^f^	1.96	0	0.65	568	17.61	513	19.49

^a^*N*,*N*-dimethylformamide, ^b^*N*,*N*-dimethylacetamide, ^c^dimethyl sulfoxide, ^d^dichloromethane, ^e^2,2,2-trifluoroethanol, ^f^1,1,1,3,3,3-hexafluoro-2-propanol, ^g^probe is insoluble in this solvent.

For both compounds, the shortest UV–vis absorption maxima were observed at λ_max_(**1-M**) = 568 nm and λ_max_(**1-P**) = 513 nm in HFIP. **1-M** shows the longest wavelength UV–vis absorption band at λ_max_ = 580 nm in DMSO, whereas **1-P** has the strongest bathochromic shift in ethanol at λ_max_ = 538 nm. These band shifts correspond to a small solvatochromic range of Δ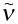 (**1-M**) = 333 cm^−1^ and Δ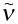 (**1-P**) = 905 cm^−1^, respectively. In general, the carbonitrile-functionalized PVAm **1-P** absorbs at shorter wavelengths as compared to the model compound **1-M**, which at firstl indicates an influence of the polymer chains on the solvatochromic behavior of the chromophoric unit.

In order to determine the relative contributions of the solvent properties on 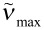, the simplified form of the Kamlet–Taft linear solvation energy relationship was used (Equation 1). The qualitatively best regressions of **1-M** and **1-P** are shown in Table 3.

**Table 3:** Solvent-independent correlation coefficients a, b and s of the Kamlet–Taft parameters *α*, *β* and π^*^; solute property of the reference system 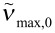, correlation coefficient (r), standard deviation (sd), number of solvents (n) and significance (f) for the solvatochromism of **1-M** and **1-P**.

comp.	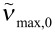 (10^3^ cm^−1^)	a	b	s	r	sd	n	f

**1-M**	17.857	0	−0.311	−0.386	0.995	0.014	10	< 0.001
**1-P**	18.260	0	−0.813	0	0.922	0.122	9	4 × 10^−4^

The correlation coefficients r are greater than 0.92 for LSERs, which indicates a high quality of the two multi-parameter equations and allows significant conclusions to be drawn. When increasing the HBA strength of the solvent, a bathochromic shift of the UV–vis absorption maxima of **1-M** is observed, which is readily explained by interaction of the solvents with the NH function of the chromophore. The negative sign of the correlation coefficient s of **1-M** indicates that the electronically excited state of these molecules becomes more strongly solvated and is consequently stabilized with increasing the solvent’s dipolarity/polarizability. This correlates with a higher dipole moment of the electronically excited state. In contrast to **1-M**, **1-P** shows no significant influence of the π^*^ term of the solvent on 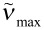, whereas the influence of the β term is greater compared to that of **1-M**. This effect reflects the impact of the NH_2_ groups of the PVAm on the UV–vis absorption maxima of the chromophore. Probably, the chains of the polymer are able to insulate the chromophores from the solvent molecules. Hence, the chromophores interact more strongly with the polymer chains than they do with the solvents. The HBD ability of the solvents shows no influence on the solvatochromic behavior of **1-M** and **1-P**.

**Figure 4:**
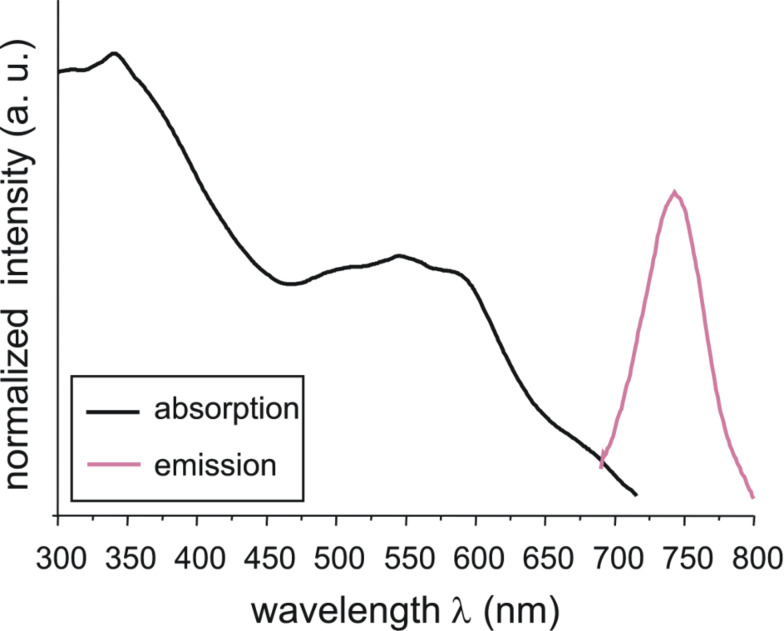
UV–vis absorption and emission diffuse reflectance spectra of sample **1-Si**.

In addition, all compounds were measured as powders by means of the diffuse reflectance technique. The UV–vis absorption bands of the solids are non-symmetric and show several UV–vis absorption maxima. The longest-wavelength UV–vis absorption maxima were observed at λ_max_ (**1-P**, **1-Si**) = 545 nm and λ_max_ (**1-M**) = 546 nm. Figure 4 shows the UV–vis absorption and the emission diffuse reflectance spectra of **1-Si**.

#### Fluorescence

Both the carbonitrile-functionalized PVAm **1-P** and the model compound **1-M** show strong fluorescence. This can be explained by two factors: Firstly, the fluorophore is a very rigid and planar molecule and secondly, the fluorophore contains both strongly electron-withdrawing and electron-donating groups along the axis of the π-conjugated system. Therefore, an efficient intramolecular charge transfer (ICT) system is possible [32]. The fluorescence emission maxima of **1-M** and **1-P** are shown in Table 4.

**Table 4:** Fluorescence emission maxima 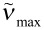 of **1-M** and **1-P**, measured in ten solvents of different polarity and hydrogen-bond ability and the corresponding Stokes shifts.

Solvent	**1-M**			**1-P**		
	λ_max,em_ (nm)	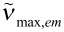 (10^3^ cm^−1^)	Stokes shift (nm)	λ_max,em_ (nm)	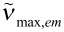 (10^3^ cm^−1^)	Stokes shift (nm)

DMF^a^	593	16.86	15	562	17.79	28
DMAA^b^	592	16.89	13	561	17.83	26
DMSO^c^	595	16.81	15	564	17.73	32
DCM^d^	584	17.06	13	— ^g^	— ^g^	—
acetonitrile	586	17.06	13	563	17.76	32
1-propanol	586	17.06	11	585	17.09	49
ethanol	587	17.04	13	586	17.06	48
methanol	588	17.01	13	582	17.18	61
TFE^e^	582	17.18	13	580	17.24	59
HFIP^f^	577	17.33	9	576	17.36	63

^a^*N*,*N*-dimethylformamide, ^b^*N*,*N*-dimethylacetamide, ^c^dimethyl sulfoxide, ^d^dichloromethane, ^e^2,2,2-trifluoroethanol, ^f^1,1,1,3,3,3-hexafluoro-2-propanol, ^g^probe is insoluble in this solvent.

**Figure 5:**
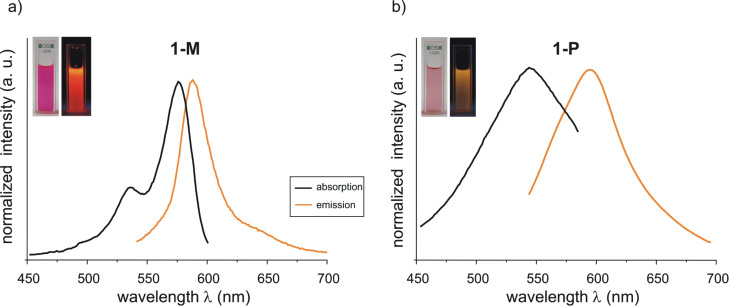
Normalized absorption and emission spectra of **1-M** (a) and **1-P** (b).

Figure 5 shows an UV–vis absorption spectrum and a fluorescence emission spectrum of **1-M** and **1-P** measured in methanol as well as a photograph of solutions of these compounds.

Similar to the UV–vis measurements, **1-M** shows the longest-wavelength emission maximum in DMSO at λ_max_ = 595 nm and **1-P** in ethanol at λ_max_ = 586 nm. The longest hypsochromic shifts of the fluorescence emission maximum were observed at λ_max_(**1-M**) = 577 nm in HFIP and λ_max_(**1-P**) = 561 nm in DMMA. These band shifts correspond to a small solvatochromic range of Δ
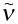
(**1-M**) = 524 cm^−1^ and Δ
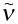
(**1-P**) = 760 cm^−1^, respectively. Furthermore, for **1-M** only a small Stokes shift (9–15 nm) is observed, whereas **1-P** shows a larger one (26–63 nm), which reaches a maximum in strong HBD solvents (HFIP).

Again, the solvent-dependent fluorescence emission maxima can be interpreted with regard to the dipolarity/polarizabilty and the hydrogen-bond capacity of the solvents using the simplified Kamlet–Taft equation (Equation 1). The qualitatively best regressions are shown in Table 5.

**Table 5:** Solvent-independent correlation coefficients a, b and s of the Kamlet–Taft parameters *α*, *β* and π^*^; solute property of the reference system 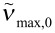, correlation coefficient (r), standard deviation (sd), number of solvents (n) and significance (f) for the fluoro-solvatochromism of **1-M** and **1-P**.

comp.	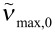 (10^3^ cm^−1^)	a	b	s	r	sd	n	f

**1-M**	17.638	0	−0.357	−0.573	0.970	0.043	10	< 0.001
**1-P**	17.465	−0.576	−0.772	0	0.922	0.144	9	0.003

**Figure 6:**
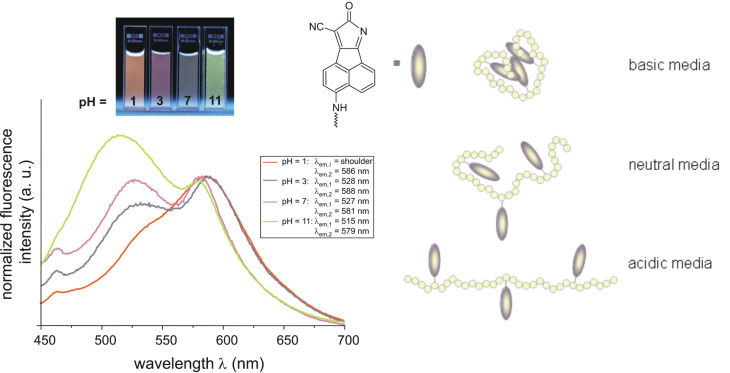
Normalized emission spectra of **1-P** in water at four different pH values and a sketch of the assumed chain conformations of PVAm.

In both, absorption and fluorescence of **1-M**, the polarity as well as the hydrogen-bond accepting ability of the solvents leads to a bathochromic band shift and both contribute to the stabilization of the excited state as well as to the interaction with the NH group. However, this effect is slightly more pronounced on the fluorescence side, as shown by the slightly higher b and s coefficients. In contrast to the results of the regression analysis obtained from the UV–vis absorption spectra, the HBD ability of the solvents shows a significant influence on the emission maxima of **1-P**. With increasing HBD strength of the solvent a bathochromic shift of the emission maximum was observed. Additionally, solvents which can act as hydrogen-bond acceptors also interact with the NH function of **1-P**, which leads to an enhancement of the push character of this group resulting in a band shift to longer wavelengths (b < 0). To confirm these conclusions drawn from the regression analysis with regard to the *α* term of the solvents, the emission spectra of **1-P** were measured in aqueous solutions at four different pH values (Figure 6).

In comparison to the UV–vis absorption measurements of the solids of **1-M**, **1-P** and the hybrid material **1-Si**, the fluorescence spectra recorded by diffuse reflection seem to be smaller (Figure 4). The model compound **1-M** exhibits an emission maximum at λ_em_ = 753 nm. For the compounds **1-Si** and **1-P** a hypsochromic band shift to λ_em_(**1-Si**) = 745 nm and λ_em_(**1-P**) = 741 nm was observed. This behavior also shows the strong influence of the chains of the PVAm on the solvatochromic behavior of the polymer.

As depicted in Figure 6, in aqueous solution at pH = 11, **1-P** shows two emission bands at λ_em,1_ = 515 nm and λ_em,2_ = 579 nm. With decreasing pH value, the intensity of the first emission band decreases, whereas for the emission band at the longer wavelength a slightly bathochromic shift is observed. This result confirms the conclusions drawn by the LSER of **1-P** with regard to the HBD strength of the solvents. A possible explanation is that at higher pH values the polymer chains are coiled and the fluorophore is not accessible for interactions with the surrounding molecules. At lower pH values the polymer has a more straight conformation and interactions of the solvent with the fluorophore are possible. The explanation is in good agreement with the known behavior of weak cationic polyelectrolyte molecules. With lowering the pH of the surrounding aqueous solution the degree of protonation increases. The increased net charge along the polymer stretches and strengthens the chain.

## Conclusion

In this paper we present the functionalization of PVAm with the known fluorophore **1** in water, mediated by 2,6-*O*-β-dimethylcyclodextrin. The characterization of the new fluorescent PVAm **1-P** has been carried out by means of ^13^C-{^1^H}-CP-MAS NMR, FTIR spectroscopy and DSC.

The influence of the solvents on the solvatochromic behavior of **1-M** and **1-P** can be quantitatively described by means of a LSE relationship using the well-established empirical Kamlet–Taft equation. The most dominant effect on the UV–vis absorption and fluorescence is caused by the HBA strength as well as by the dipolarity/polarizabilty of the solvents. In particular it has been shown that the polymer chains have a significant influence on the solvatochromic behavior of **1-P**. With decreasing pH value in aqueous solutions a bathochromic band shift was observed.

Pre-adsorption of carbonitrile **1** on silica and subsequent nucleophilic substitution of PVAm in water at 100 °C results in fluorophore-functionalized PVAm/silica particles. The PVAm–carbonitrile layer remains irreversibly bound to the silica, as shown by extraction experiments. Hence, the synthesis of a fluorescent hybrid material was possible.

## Experimental

*General details:* The aqueous solution of poly(vinyl amine) copolymer (*M*_n_ = 15,000 g mol^−1^, pH = 11) was kindly provided by BASF SE (Ludwigshafen, Germany). 2,6-*O*-β-dimethylcyclodextrin (β-DMCD) was supplied by Wacker Chemie (Burghausen, Germany).

### 2-(2-Oxo-2*H*-acenaphthylene-1-ylidene)-malononitrile (**2**)

Compound 2 was synthesized as described earlier [34]. Acenaphthene-1,2-dione (2.00 g, 11 mmol) and malononitrile (0.73 g, 11 mmol) were dissolved in 40 mL of CH_3_CN and refluxed for 3 h. After cooling, the orange precipitate was filtered off and washed several times with CH_3_CN to yield **2** as an orange crystalline solid (2.00 g, 8.3 mmol). Yield 80%, mp 241–244 °C (lit. 243–245 °C); ^1^H NMR (250 MHz, CD_2_Cl_2_): δ (ppm) 8.61 (dd, *J* = 7.5 Hz, *J* = 0.6 Hz, 1H), 8.33–8.29 (m, 2H), 8.21 (dd, *J* = 7.2 Hz, *J* = 0.8 Hz, 1H), 7.96–7.88 (m, 2H); ^13^C NMR (69 MHz, CD_2_Cl_2_): δ (ppm); 142.9, 132.6 (Ar*H*), 132.3 (Ar*H*), 130.5, 128.3, 112.7, 129.2 (Ar*H*), 129.0 (Ar*H*), 124.5 (Ar*H*), 123.6 (Ar*H*); FTIR (KBr): 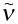 (cm^−1^) 3093, 2225, 1717, 1597, 1574; C_15_H_6_N_2_O (230.23) Anal. calcd. C, 78.26; H, 2.63; N, 12.17; found C, 78.03; H, 2.66; N, 12.18.

### 8-Oxo-8*H*-acenaphtho[1,2-*b*]pyrrol-9-carbonitrile (**1**)

2-(2-Oxo-2*H*-acenaphthylene-1-ylidene)-malononitrile (**2**) (0.80 g, 3.5 mmol) and K_2_CO_3_ (48 mg, 0.35 mmol) in 5 mL of CH_3_CN were refluxed for 1 h. After cooling, the yellow-orange precipitate was filtered off and washed several times with CH_3_CN to yield **1** as a yellow–orange solid (0.70 g, 3.1 mmol). Yield 88%, mp 278–280 °C (lit. 275–277 °C [32]); ^1^H NMR (250 MHz, CD_2_Cl_2_): δ (ppm) 8.80 (dd, *J* = 7.4 Hz, *J* = 1.3 Hz, 1H), 8.51–8.41 (m, 3H), 7.99 (dd, *J* = 7.6 Hz, *J* = 0.5 Hz, 1H), 7.89 (dd, *J* = 8.3 Hz, *J* = 1.0 Hz, 1H); FTIR (KBr): 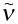 (cm^−1^) 3084, 2231, 1713, 1645, 1576, 1549; C_15_H_6_N_2_O (230.23) Anal. calcd. C, 78.26; H, 2.63; N, 12.17; found C, 77.96; H, 2.61; N, 11.79.

### Fluorophoric, carbonitrile-functionalized PVAm (**1-P**)

For the β-cyclodextrin complex formation, stoichiometric amounts of **1** (0.29 g, 1.28 mmol) and β-DMCD (1.68 g, 1.28 mmol) were dissolved in methanol and stirred overnight at room temperature [29]. The light orange solid complex of **1-DMCD** was isolated by removing the methanol in vacuum and was used without further purification.

For functionalization of PVAm in aqueous media, the solid β-DMCD complexes (10 mol %) was dissolved in 50 mL of distilled water and 6.10 g of the aqueous solution of PVAm containing 0.50 g of the polymer added. The mixture was refluxed for 8 h. Then the fluorophore-functionalized PVAm was precipitated by addition to ice-cold acetone (refrigerator), washed with acetone and dried in vacuum to yield an intensive purple-colored solid.

^13^C-{^1^H}-CP-MAS NMR (100 MHz, 12.5 kHz): δ (ppm) 181.3, 168.6, 128.4, 71.5, 46.0, 31.6, 23.5; FTIR (KBr): 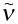 (cm^−1^) 3239, 2895, 2838, 2221, 1665, 1590, 1497, 1386; Anal. calcd (for 100% substitution). C, 55.73; H, 10.11; N, 31.20; found C, 57.27; H, 7.45; N, 15.71.

### 3-Isopropylamino-8-oxo-8*H*-acenaphtho[1,2-*b*]pyrrol-9-carbonitrile (**1-M**)

8-Oxo-8*H*-acenaphtho[1,2-*b*]pyrrol-9-carbonitrile (**1**) (88 mg, 0.38 mmol) and isopropylamine (45 mg, 0.76 mmol) in 15 mL of CH_3_CN were stirred for 3 h at room temperature. The dark purple-colored precipitate was filtered off and washed several times with CH_2_Cl_2_ to yield **1-M** (51 mg, 18 mmol).

Yield 47%, mp > 300 °C (lit. >300 °C [33]); ^1^H NMR (250 MHz, CD_2_Cl_2_): δ (ppm) 8.41 (d, 1H, *J* = 7.7 Hz), 8.15 (t, *J* = 7.6 Hz, *J* = 9.4 Hz, 2H), 7.69 (t, *J* = 7.6 Hz, *J* = 7.7 Hz, 1H), 7.36 (d, *J* = 9.4 Hz, 1H), 4.22–4.31 (m, 1H) NHC*H*) 1.49–1.51 (m, 6H, CH(C*H*_3_)_2_; ^13^C-{^1^H}-CP-MAS NMR (100 MHz, 12.5 kHz): δ (ppm) 185.3, 174.7, 171.8, 154.3, 137.9, 130.0, 1278.0, 124.8, 123.5, 121.9, 118.7, 117.3, 115.5, 111.6, 107.3, 104.8, 48.2, 23.2, 22.2; FTIR (KBr): 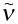 (cm^−1^) 3334, 3049, 2982, 2213, 1627, 1576, 1539; C_18_H_13_N_3_O (287.32) Anal. calcd. C, 75.25; H, 4.56; N, 14.63; found C, 74.85; H, 4.37; N, 14.49.

### Fluorophoric, carbonitrile-functionalized PVAm/silica particles (**1-Si**)

For functionalization of silica with fluorophores and PVAm in aqueous media, **1** (0.1 g, 0.4 mmol) was dissolved in 50 mL of CH_2_Cl_2_, then suspended with silica (2.5 g, 41 mmol) and kept overnight. The solvent was removed in a rotary evaporator at 40 °C. Distilled water (60 mL) and 2.2 g of the aqueous solution of PVAm (0.18 g of polymer, 4.1 mmol) were added to the freshly prepared **1**-loaded silica particles. The mixture was refluxed for 16 h. After cooling to room temperature, the PVAm-functionalized silica was filtered off and washed carefully with water. In order to remove non-adsorbed functionalized PVAm and unreacted **1** from the silica particles surface, two Soxhlet extraction cycles were carried out, the first one with water and the second with acetone. **1-Si** was obtained as purple solid.

^13^C-{^1^H}-CP-MAS NMR (100 MHz, 12.5 kHz): δ (ppm) 181.2, 164.7, 151.4, 128.3, 44.6, 37.4; FTIR (KBr): 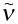 (cm^−1^) 3277, 2207, 1651, 1572, 1569; 1087, 1025; Anal. calcd. C, 6.30; H, 0.82; N, 2.52; found C, 5.61; H, 1.24; N, 2.08.
